# Nur77 Deficiency Exacerbates Macrophage NLRP3 Inflammasome-Mediated Inflammation and Accelerates Atherosclerosis

**DOI:** 10.1155/2022/2017815

**Published:** 2022-04-13

**Authors:** Ruosen Yuan, Weifeng Zhang, Peng Nie, Keke Lan, Xiaoxiao Yang, Anwen Yin, Qingqing Xiao, Yejiao Shen, Ke Xu, Xia Wang, Xin Pan, Linghong Shen, Ben He

**Affiliations:** ^1^Department of Cardiology, Shanghai Chest Hospital, Shanghai Jiao Tong University, Shanghai 200030, China; ^2^Department of Cardiology, Shanghai Jiao Tong University Affiliated Sixth People's Hospital, Shanghai, China; ^3^Department of Cardiology, Renji Hospital, Shanghai Jiaotong University School of Medicine, Shanghai, China

## Abstract

**Purpose:**

Activation of NLR (nucleotide-binding and leucine-rich repeat immune receptor) family pyrin domain containing 3 (NLRP3) inflammasome mediating interleukin- (IL-) 1*β* secretion has emerged as an important component of inflammatory processes in atherogenesis. The nuclear receptor Nur77 is highly expressed in human atherosclerotic lesions; however, its functional role in macrophage NLRP3 inflammasome activation has not yet been clarified. *Methods, Materials, and Results*. Eight-week-old apolipoprotein E (ApoE)−/− and ApoE−/− Nur77−/− mice that were fed a Western diet underwent partial ligation of the left common carotid artery (LCCA) and left renal artery (LRA) to induce atherogenesis. Four weeks later, severe plaque burden associated with increased lipid deposition, reduced smooth muscle cells, macrophage infiltration, and decreased collagen expression was identified in ApoE−/− Nur77−/− mice compared with those in ApoE−/− mice. ApoE−/− Nur77−/− mice showed increased macrophage inflammatory responses in carotid atherosclerotic lesions. In vitro studies demonstrated that oxidized low-density lipoprotein cholesterol (ox-LDL) increased the release of lactate dehydrogenase (LDH) and upregulated the expressions of cleaved caspase-1, cleaved IL-1*β* and gasdermin D (GSMD) in WT peritoneal macrophages (PMs) in a NLRP3-dependent manner. Nur77−/− PMs exhibited a further increased level of NLRP3 inflammasome-mediated inflammation under ox-LDL treatment compared with WT PMs. Mechanistically, Nur77 could bind to the promoter of NLRP3 and inhibit its transcriptional activity.

**Conclusions:**

This study demonstrated that Nur77 deletion promotes atherogenesis by exacerbating NLRP3 inflammasome-mediated inflammation.

## 1. Introduction

Atherosclerosis is a chronic inflammatory disease that arises from an imbalance in lipid metabolism and a maladaptive immune response and considered as the leading cause of morbidity and mortality worldwide [1]. Rupture of atherosclerotic plaques results in thrombotic occlusion of blood vessels, inducing the clinical cardiovascular events like myocardial and cerebral infarction [2]. It is well known that blood monocytes are recruited to the inflamed vascular wall to differentiate into inflammatory macrophages (M1-like phenotype) and foam cells, which contribute to pathogenesis at many stages of atherosclerosis [3]. In addition to hyperlipidemia and other risk factors (e.g., hypertension, diabetes mellitus, and smoking), inflammation is now increasingly recognized as a critical contributor to atherogenesis [4]. The activation of nucleotide-binding and leucine-rich repeat immune receptor (NLR) family pyrin domain containing 3 (NLRP3) inflammasome mediating interleukin- (IL-) 1*β* secretion has recently emerged as an important component of inflammatory processes underlying atherosclerosis [5]. Uncovering the regulation mechanisms of the process may provide a novel avenue for atherosclerosis treatment.

The NLRP3 inflammasome is a multiprotein platform consisting of NLRP3, apoptosis-associated speck-like protein containing a caspase recruitment domain (ASC), and the cysteine protease caspase-1 [6]. Upon activation, NLRP3 forms an inflammasome complex with ASC and causes a conversion of pro-caspase-1 to active caspase-1, which subsequently leads to the processing and secretion of mature IL-1*β* and IL-18 [7]. IL-1*β* is an important proatherogenic factor, and a recent study revealed that targeting IL-1*β* using an IL-1*β*-neutralizing antibody has proven beneficial for cardiovascular diseases in the Canakinumab Anti-Inflammatory Thrombosis Outcomes Study (CANTOS) trial [8]. Since the NLRP3 inflammasome acts as a central producer of IL-1*β* [5], understanding the regulation of NLRP3 inflammasome activation has important therapeutic implications. Although the upstream mechanism of NLRP3 inflammasome activation has been elaborated [5, 7], the exact downstream mechanism by which NLRP3 is regulated is not yet completely understood.

Nuclear receptor Nur77, also known as NR4A1, TR3, or NGFI-B, is a member of the NR4A receptor subfamily that also comprises Nurr-1 (NR4A2) and NOR-1 (NR4A3) [9]. They are referred as “orphans” because their ligands are currently not identified [10]. In previous studies, Nur77 expression could be induced in macrophages by several stimuli related to development of atherosclerosis such as oxidized low-density lipoprotein (ox-LDL), lipopolysaccharide (LPS), and tumor necrosis factor-*α* (TNF-*α*) [11]. And Nur77 deficiency exacerbates a wide variety of inflammatory diseases, including atherosclerosis [12]. Previous studies have shown that Nur77 plays a crucial role in the functional regulation of cell proliferation, differentiation, and apoptosis [13]. Recent interest has focused on its robust anti-inflammatory effect in inflammation-driven pathologies, and genetic studies subsequently reported an essential role of Nur77 in controlling the inflammatory responses in atherogenesis [14, 15]. However, whether Nur77 elicits vascular protective effects through regulating NLRP3 inflammasome is undetermined.

Therefore, in this study, we induced apolipoprotein E (ApoE)−/− Nur77−/− mice, and combined partial ligation of left common carotid artery (LCCA) and left renal artery (LRA) was conducted. We aimed to investigate whether Nur77 has a function role in macrophage NLRP3 inflammasome-mediated inflammation.

## 2. Methods and Materials

### 2.1. Mice

All experimental procedures described conformed to the National Institutes of Health Research guidelines for animal experimentation and were approved by our institutional ethics committee. ApoE−/− and ApoE−/− Nur77−/− mice (C57BL/6J background), used in this study, were obtained from the Jackson Laboratory. Female mice (7 weeks old) were allowed to adapted to the environment for one week, and a Western diet was started and continued for 6 weeks. As previously described [16], eight-week-old mice received a combined partial ligation of LCCA and LRA. A sham operation was performed in the Ctrl group. The cervical regions of mice were scanned by MRI 4 weeks after the surgery and then the mice were euthanized. The carotid arteries were isolated and embedded in OCT compound for further analysis. All of the procedures were carried out under an anatomic microscope.

### 2.2. Cell Culture

Peritoneal macrophages (PMs) were used in this study. PMs were obtained from both wild-type (WT) and Nur77−/− mice as previously described [17]. Mice were intraperitoneally injected with 2 ml 4% thioglycolate broth per mouse, and 4 days later, the cells were collected by peritoneal lavage with 15 ml of cold PBS. PMs with purity >95% were cultured in DMEM medium containing 10% FBS with 5% CO_2_ at 37°C and were used for subsequent experiments.

### 2.3. Western Blotting

The cells were lysed with lysis buffer, separated by SDS-PAGE, and transferred to polyvinylidene difluoride membrane. Membranes then were blocked with 5% nonfat milk and incubated with primary antibody, followed by detection using an enhanced chemiluminescence system. The following primary antibodies were used: anti-NLRP3 (Cat# ab214185, Abcam), anti-caspase-1 (Cat# 3866, Cell Signaling Technology), anti-cleaved caspase-1 (Cat# 3866, Cell Signaling Technology), anti-IL-1*β* (Cat# AB41610, Absci), anti-cleaved IL-1*β* (Cat# AB41610, Absci), anti-GSMD (Cat# 39754, Cell Signaling Technology), anti-cleaved GSMD (Cat# 39754, Cell Signaling Technology), and anti-Nur77 (Cat# 3960, Cell Signaling Technology).

### 2.4. Cell Viability

Cell viability was measured by lactate dehydrogenase (LDH) release assay (Cat# C0016, Beyotime), according to the manufacturer's instructions. The culture supernatants were collected and centrifuged at 12000 g for 5 min to remove cell debris. LDH release in the supernatants was measured at OD 490.

### 2.5. RNA Extraction and Quantitative Real-Time PCR

Total RNA was isolated from macrophages using TRIzol reagent (Life Technologies). RNA (2 *μ*g) was used to create the first-strand cDNA, and quantitative reverse transcription-polymerase chain reaction (RT-PCR) was performed using SYBR Green dye and the Roche LightCycler® 480 II system. The relative mRNA expression levels of NLRP3 and IL-1*β* were normalized to *β*-actin. Primer sequences for *β*-actin, NLRP3, and IL-1*β* were shown as follows: *β*-actin, forward 5′-TGTA CCCA GGCA TTGC TGAC-3′, reverse 5′-AACG CAGC TCAG TAAC AGTCC-3′; NLRP3, forward 5′-GTCA CCAT GGGT TCTG GTCA-3′, reverse 5′-GGGC TTAG GTCC ACAC AGAA-3′; and IL-1*β*, forward 5′-TGCC ACCT TTTG ACAG TGATG-3′, reverse 5′-TGAT GTGC TGCT GCGA GATT-3′.

### 2.6. Dual-Luciferase Assay

To measure the activity of NLRP3 reporter, Raw264.7 cells were seeded into 24-well plates. After 12 h, following the manufacturer's instructions, Nur77 and dual-luciferase reporter plasmids were transiently transfected into the cells using Lipofectamine 3000 (Invitrogen). 24 hours later, cell lysates were prepared by incubating cells in 1× lysis buffer (Luciferase Assay System, Promega, Madison, WI, USA) for 10 mins. Finally, firefly and renilla luciferase activity levels were tested according to the manufacturer's dual-luciferase assay protocol.

### 2.7. CHIP Assay

Chromatin immunoprecipitation (ChIP) assays were performed with SimpleChIP® Enzymatic Chromatin IP Kit (Cat# 9003, Cell Signaling Technology) according to the manufacturer's instructions. Raw264.7 cells were treated with PBS or ox-LDL (100 *μ*g/ml) for 24 h and then crosslinked with 1% formaldehyde for 20 mins at room temperature. After termination using the addition of glycine, crosslinked chromatin was digested with Micrococcal Nuclease, and sheared chromatin was then immunoprecipitated with anti-Nur77 (Cat# NB100-56745, Novus Biologicals) or anti-IgG antibody, respectively. Immunoprecipitated chromatin was decrosslinked at 65°C for 4 h and purified by using spin columns. ChIP PCR was performed with primers encompassing the loci of the NLRP3 promoter in mice: forward: 5′-CCAT GGTC AGAC AGTG GTTCT-3′ and reverse: 5′-CCAT GACT TTTG GTCT CACCTG-3′. Then, NLRP3 promoter-specific PCR products were subjected to agarose gel electrophoresis analysis.

### 2.8. Immunofluorescence

As previously described [18], frozen sections were fixed with 4% paraformaldehyde, washed with phosphate buffer saline (PBS), permeabilized with 0.2% Triton X-100, and blocked with 5% FBS, followed by incubation with primary antibodies anti-CD68 (Cat# ab53444, Abcam), anti-smooth muscle cell (SMC) *α*-actin (Cat# ab21027, Abcam), anti-cleaved caspase-1 (Cat# 3866, Cell Signaling Technology), anti-cleaved IL-1*β* (Cat# AB41610, Absci), and anti-Nur77 (Cat# ab153914, Abcam) overnight. The samples were then incubated with secondary antibodies and counterstained with 4′,6-diamidino-2-phenylindole. Fluorescent images were finally taken with a Zeiss LSM laser confocal scanning microscope within 24 hours.

### 2.9. Tissue Collection and Processing

Mice were euthanized by an overdose of carbon dioxide and then perfused via the left ventricle as follows: 10 ml PBS, 10 ml 4% paraformaldehyde, and 10 ml PBS. The common carotid arteries were embedded and snap-frozen in O.C.T. Tissue Tek Compound (Sakura Finetek, Torrance, CA, USA) and then stored at −80°C until use. As previously described [16], serial cross-sections (6*μ*m) were cut every 200 *μ*m over the full length of the LCCA.

### 2.10. Histology

The samples were stained with hematoxylin and eosin (HE), Sirius red, and Oil red O according to the standard protocols. Images were captured under a DP71 digital camera mounted on an Olympus CX41 compound microscope (Tokyo, Japan). The plaque burden and the content of lipid deposition and collagen were analyzed using ImagePro Plus software (Media Cybernetics, Rockville, MD, USA).

### 2.11. Blood Biochemistry

Mice were anesthetized and the blood samples were collected from the right retroorbital plexus of mice. The serum was separated by centrifugation with 3500 × *g* at 4°C for 15 min and then stored at −80°C until use. According to the standard protocols, the levels of creatinine, alanine aminotransferase (ALT), total cholesterol, low-density lipoprotein cholesterol (LDL), high-density lipoprotein cholesterol (HDL), and triglycerides were analyzed using a Hitachi 7180 autoanalyzer (Hitachi High-Technologies Corp, Japan).

### 2.12. Morphometry

In order to analyze several plaque parameters (e.g., plaque burden, SMC *α*-actin, macrophage, Sirius red, lipid deposition, cleaved-caspase-1, and cleaved-IL-1*β*), cryosections equally spaced at 100 *μ*m intervals over the common carotid artery were used for analysis. For morphometric data, 8 sections were analyzed per mouse. As previously described [19], the positive areas of SMC *α*-actin, macrophage, Sirius red, Oil red O, cleaved-caspase-1, and cleaved-IL-1*β* were calculated using ImagePro Plus software (Media Cybernetics, Rockville, MD, USA) and normalized to the plaque area.

### 2.13. Statistical Analysis

Data are expressed as mean ± standard error of the mean (SEM). Statistics normality of data was checked using the Shapiro-Wilk normality test. Statistical analysis among groups were performed with either Student's *t* test (two groups) or one-way analysis of variance (ANOVA) followed by Bonferroni's test (≥ three groups). Differences in the classification and occurrence of adverse events were analyzed with *χ*^2^ tests. *P* values < 0.05 were considered as statistically significant ^∗^*P* < 0.05. Unless otherwise noted, all data analysis was conducted with GraphPad Prism version 7.0.

## 3. Result

### 3.1. Nur77 Deficiency Accelerates Plaque Formation in ApoE−/− Mice

**Figure 1:**
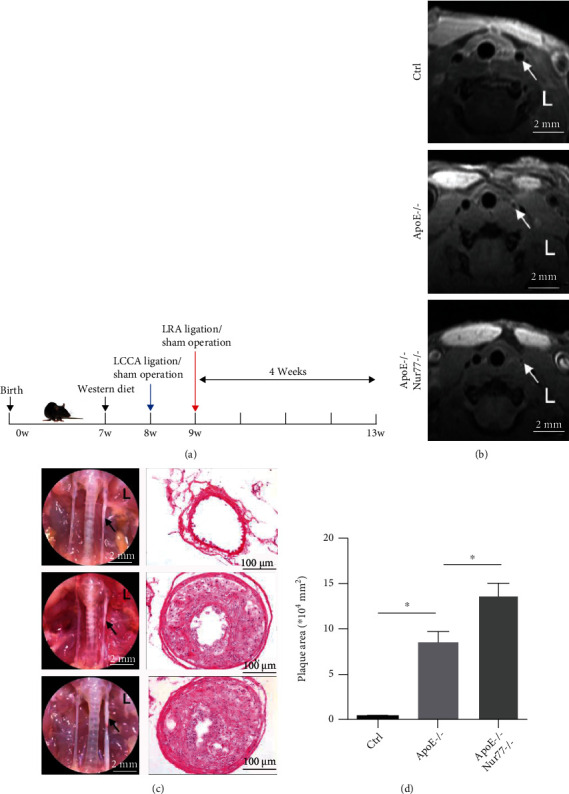
Nur77 deficiency promotes atherosclerotic plaque progression and destabilization. (a) Schematic illustration of the study design of the timeline and treatment protocol. (b) Representative magnetic resonance images of the cross-sections of LCCA in mice from each group 4 weeks after the surgeries (scale bar = 2 mm). The vascular lumen was shown by a white arrow. (c) Representative gross images of LCCA segments (scale bar = 2 mm) and HE staining of LCCA sections in mice from each group (scale bar = 100 *μ*m). (d) Quantification of the carotid atherosclerotic plaque areas in mice from each group (*n* = 12–17). Values are shown as the mean ± SEM. LCCA: left common carotid artery; LRA: left renal artery; L: left. ^∗^*P* < 0.05.

**Figure 2:**
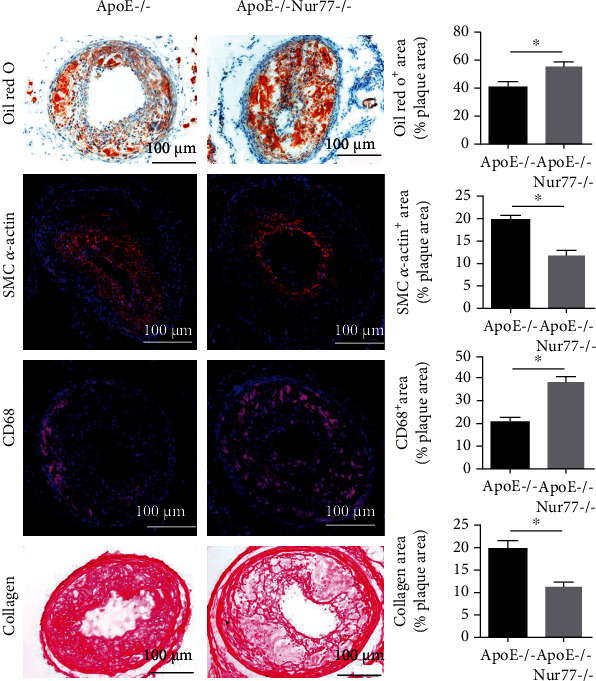
Effect of Nur77 deficiency on the cellular composition of atherosclerotic lesions in ApoE−/− mice. Representative images of carotid atherosclerotic lesion composition in mice from each group by staining with Oil red O, smooth muscle cell *α*-actin (SMC *α*-actin), CD68, or collagen. Quantification of Oil red O+, SMC *α*-actin+, CD68+, and collagen+, areas relative to the total plaque area. Scale bar = 100 *μ*m; *n* = 8. Values are shown as the mean ± SEM. ^∗^*P* < 0.05.

To investigate the impact of Nur77 on atherogenesis, ApoE−/− and ApoE−/− Nur77−/− mice received a sham operation or combined partial ligation of LCCA and LRA (Figure 1(a)). After 4 weeks of surgery, the mice were scanned using MRI and then were euthanized. The plaque burden and cellular composition of the lesions were determined in LCCA sections. The representative MRI images of LCCA were shown (Figure 1(b)). As shown in Figure 1(c), the ApoE−/− group showed stable and increased intimal area compared with the Ctrl group. Unstable plaques were observed in ApoE−/− Nur77−/− mice and the lesion size increased nearly 1.59 times compared with that in the ApoE−/− group (Figure 1(d)). A strikingly increased intimal area was observed in ApoE−/− Nur77−/− mice compared with that in the ApoE−/− group (*P* < 0.05). Further characterization of the lesion phenotype showed that 90% (9/10) of lesions in ApoE−/− Nur77−/− group and 40% (4/10) of lesions in ApoE−/− group had vulnerable features, accompanied by an increasing trend of multiple layers with discontinuity (70% vs. 30%), indicative of plaque instability (Table 1). To characterize the cellular composition of the lesion, we performed immunohistochemical staining and observed increased macrophages and lipid content in lesions from ApoE−/− Nur77−/− mice compared with those from the ApoE−/− group (*P* < 0.05) (Figure 2). Moreover, lesions of ApoE−/− Nur77−/− mice contained less SMC *α*-actin and collagen content than lesions from the ApoE−/− group (*P* < 0.05) (Figure 2). No significant differences were observed in the levels of total cholesterol, LDL, HDL, triglycerides, creatinine, and ALT between the ApoE−/− and ApoE−/− Nur77−/− groups (Fig. S1).

### 3.2. Nur77 Deficiency Exacerbates the Inflammatory Responses in Atherosclerotic Plaques

**Figure 3:**
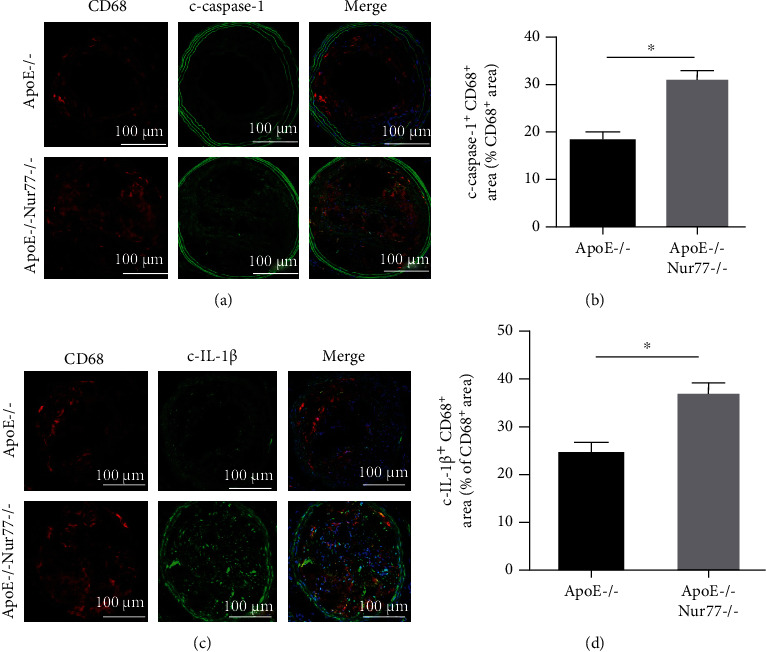
Effect of Nur77 deficiency on macrophage inflammatory responses of atherosclerotic lesions. (a) Representative immunofluorescence staining of CD68 (red), c-caspase-1 (green), and their colocalization (yellow) of carotid atherosclerotic lesions in mice from each group (scale bar = 50 *μ*m). (b) Quantification of c-caspase-1+ CD68+ areas relative to the total CD68+ area (*n* = 8). (c) Representative immunofluorescence staining of CD68 (red), c-IL-1*β* (green), and their colocalization (yellow) of carotid atherosclerotic lesions in mice from each group (scale bar = 50 *μ*m). (d) Quantification of c-IL-1*β*+ CD68+ areas relative to the total CD68+ area (*n* = 8). Values are shown as the mean ± SEM. ^∗^*P* < 0.05.

IL-1*β* is an important vascular and systemic inflammatory mediator that is regulated by NLRP3 inflammasome and contributes to atherogenesis [5]. To examine the effect of Nur77 on IL-1*β*-induced inflammation in atherosclerotic plaques, we evaluated the expression levels of cleaved caspase-1 (c-caspase-1) and cleaved IL-1*β* (c-IL-1*β*). Immunofluorescence colocalization of c-caspase-1 and macrophage showed that c-caspase-1+ staining of CD68+ macrophages in carotid lesions was significantly increased in the ApoE−/− Nur77−/− group compared with that in the ApoE−/− group (*P* < 0.05) (Figures 3(a) and 3(b)). Further, we observed that the c-IL-1*β*+ staining of CD68+ macrophages in atherosclerotic plaques was also markedly upregulated in ApoE−/− Nur77−/− group compared with that in the ApoE−/− group (*P* < 0.05) (Figures 3(c) and 3(d)). These results reveal that Nur77 deletion promotes macrophage inflammatory responses of atherosclerotic lesions.

### 3.3. ox-LDL Activates NLRP3 Inflammasome-Mediated Inflammation in PMs

**Figure 4:**
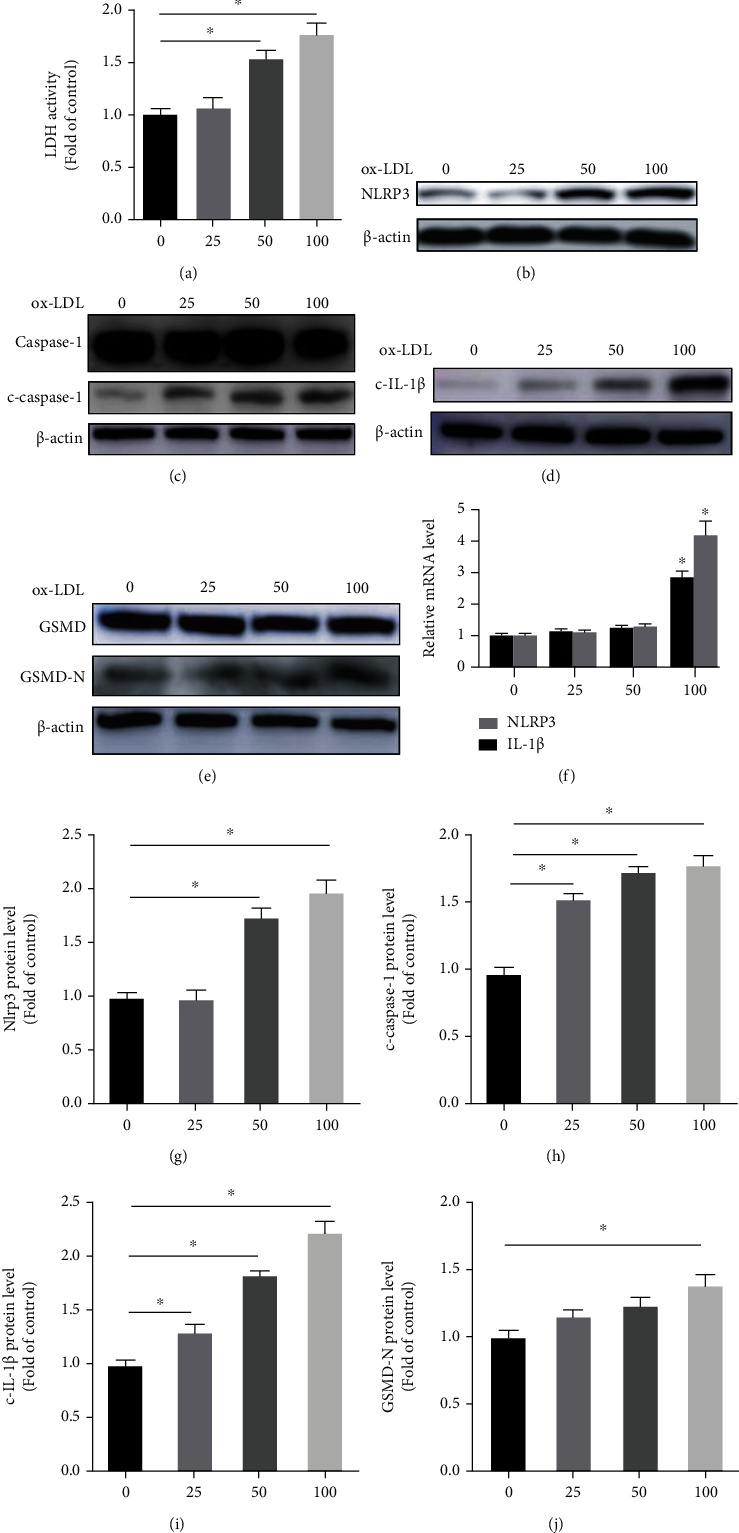
ox-LDL activates NLRP3 inflammasome and induces IL-1*β*-mediated inflammation in PMs. PMs were treated with ox-LDL at a gradient concentration of 0, 25, 50, and 100 *μ*g/ml for 24 h and then were collected for analysis. (a) The LDH activity in supernatant was assessed by LDH Cytotoxicity Assay Kit. The expression levels of NLRP3 inflammasome-related protein NLRP3 (b), c-caspase-1 (c), c-IL-1*β* (d), and GSMD-N (e) were determined by Western blotting. (f) The relative mRNA level of NLRP3 and IL-1*β*; ∗*P* <0.05 compared with the control group. Relative protein levels of NLRP3 (g), c-caspase-1 (h), c-IL-1*β* (i) and GSMD-N (j). n = 3. Values are shown as the mean ± SEM. ∗*P* <0.05.

To explore the mechanism of the inflammation activated by Nur77 deletion in atherosclerotic lesions, we firstly detected the change of NLRP3 inflammasome in cultured PMs. The primary PMs were stimulated with a gradient concentration of ox-LDL, which was considered as a vital proatherosclerotic factor. Cell viability was assessed by measuring the release of LDH, and the results showed that LDH activity increased with ox-LDL treatment in a dose-dependent manner (Figure 4(a)). We next performed Western blot to detect the target proteins: NLRP3, precursors of caspase-1 (pro-caspase-1), c-caspase-1, and c-IL-1*β*. As shown in Figures 4(b) and 4(g), the protein expression of NLRP3 under the stimulation of high concentration of ox-LDL was significantly upregulated as compared to those in the control group. Meanwhile, the expression levels of the c-caspase-1 and c-L-1*β* were also strikingly upregulated in PMs after ox-LDL treatment in a dose-dependent manner (Figures 4(c), 4(d), 4(h), and 4(i)). Recent studies have reported that c-caspase-1 function as not only processing the pro-IL-1*β* into their mature forms but also mediating proteolytic cleavage of gasdermin D (GSMD), which is responsible for inflammasome-induced inflammatory cell death termed pyroptosis [5, 20]. We then evaluated the pyroptosis indicators such as GSMD and GSMD-N domains (GSMD-N), and the results showed that the expression level of GSMD-N was significantly increased only after high concentration of ox-LDL treatment (Figures 4(e) and 4(j)). Finally, quantitative RT-PCR results showed that the transcription levels of NLRP3 and IL-1*β* were substantially elevated after treatment with high concentration of ox-LDL (Figure 4(f)).

**Figure 5:**
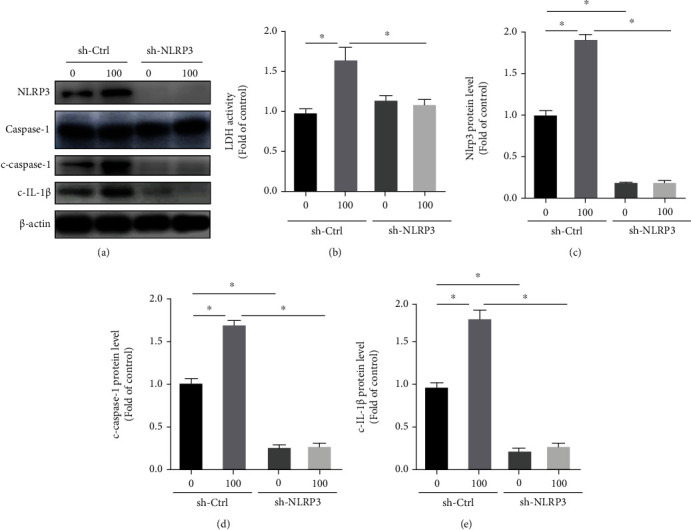
NLRP3 knockdown suppresses ox-LDL-induced IL-1*β*-mediated inflammation and NLRP3 inflammasome activation in PMs. PMs were transfected with sh-Ctrl or sh-NLRP3 for 24 hours, treated with or without ox-LDL (100 *μ*g/ml) for another 24 h, and then were collected for analysis. (a) Western blot analysis of NLRP3 inflammasome-related proteins. (b) The LDH activity in supernatant was assessed by LDH Cytotoxicity Assay Kit. Relative protein levels of NLRP3 (c), c-caspase-1 (d), and c-IL-1*β* (e). *n* = 3. Values are shown as the mean ± SEM. ^∗^*P* < 0.05.

To further examine whether ox-LDL-induced macrophage inflammation was NLRP3 dependent, we regulated the expression of NLRP3 using shRNA targeting NLRP3 (Figures 5(a) and 5(c)). We found that silence of NLRP3 dampened the expressions of c-caspase-1 and c-IL-1*β* induced by ox-LDL (Figures 5(a), 5(d), and 5(e)). Moreover, we also found that silencing NLRP3 abolished the release of LDH from PMs after ox-LDL treatment (Figure 5(b)), indicating that NLRP3 inflammasome was at least partly responsible for the death of PMs. All these findings indicate that ox-LDL induces NLRP3 inflammasome-mediated inflammatory responses in PMs.

### 3.4. Nur77 Suppresses NLRP3 Inflammasome Activation by Transcriptionally Inhibiting NLRP3

**Figure 6:**
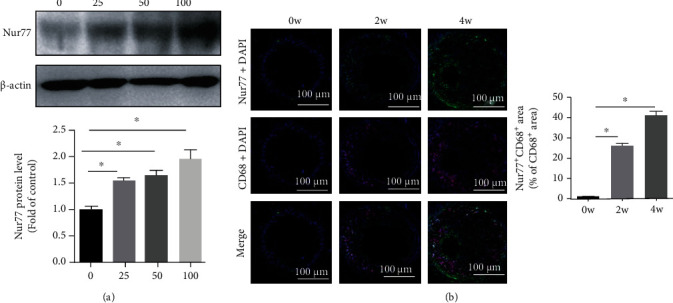
Expression of Nur77 in PMs in vivo and in vitro in response to ox-LDL. (a) PMs were treated with increasing concentrations of ox-LDL for 24 h, and the protein expression of Nur77 was examined by Western blotting (*n* = 3). (b) Representative images of dual immunofluorescence staining of Nur77 (green) and CD68 (red) and quantification of the ratio of Nur77-positive area to CD68-positive area (*n* = 8). Values are shown as the mean ± SEM. ^∗^*P* < 0.05.

We firstly examined the expression of Nur77 in cultured PMs in response to ox-LDL stimulation. The results demonstrated that the protein expression of Nur77 upregulated in a dose-dependent manner after ox-LDL stimulation (Figure 6(a)). Consistently, the Nur77+ staining of CD68+ macrophages in atherosclerotic plaques was also markedly increased with the extension of time in the model group compared with that in the control group (Figure 6(b)).

**Figure 7:**
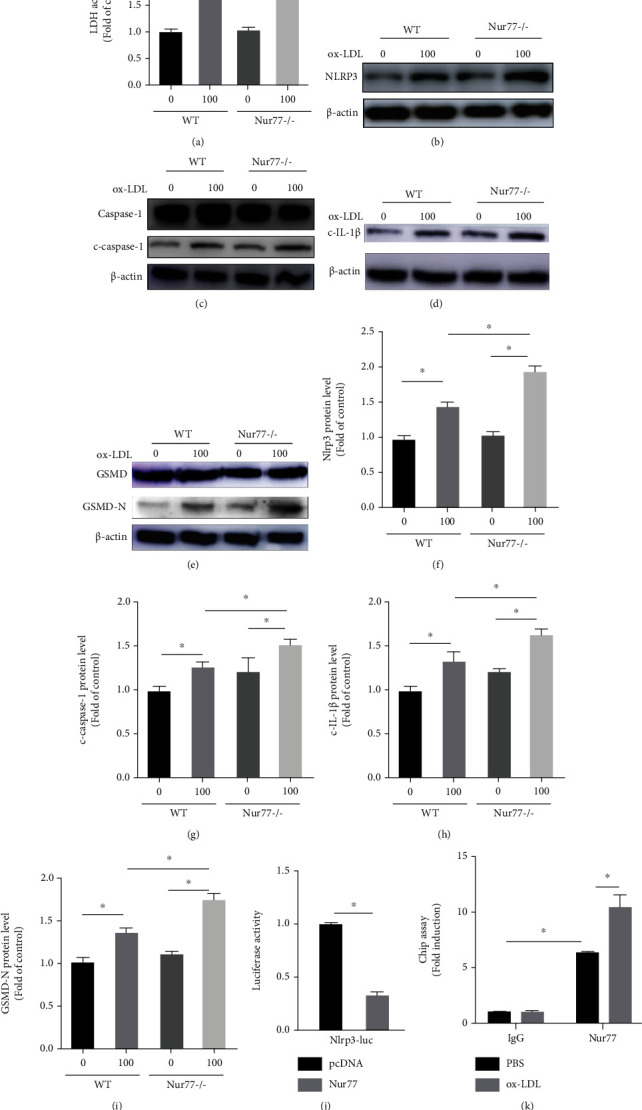
Nur77 deficiency exacerbates NLRP3 inflammasome-mediated inflammation in PMs. PMs from WT or Nur77−/− mice were stimulated with or without ox-LDL (100 *μ*g/ml) for 24 h and then were collected for analysis. (a) The LDH activity in supernatant was assessed by LDH Cytotoxicity Assay Kit. The expression levels of NLRP3 inflammasome-related proteins NLRP3 (b), c-caspase-1 (c), c-IL-1*β* (d), and GSMD-N (e) were determined by Western blotting. Relative protein levels of NLRP3 (f), c-caspase-1 (g), c-IL-1*β* (h), and GSMD-N (i). (j) The mice NLRP3 promoter reporters, blank PGL3, and pcDNA or Nur77 plasmid were transiently transfected into Raw264.7 cells; after 24 h, the dual-luciferase activity was measured. (k) Raw264.7 cells were treated with or without ox-LDL (100 *μ*g/ml) for 24 h; DNA fragments from the Raw264.7 cells that contain regions of the NLRP3 promoter were immunoprecipitated with the anti-Nur77 antibody. The expression was identified by q-PCR. *n* = 3. Values are shown as the mean ± SEM. ^∗^*P* < 0.05.

To determine the role of Nur77 in ox-LDL-induced PM NLRP3 inflammasome activation, Nur77−/− PMs and WT PMs were isolated from Nur77−/− mice and WT mice, respectively. The test of the supernatant LDH showed that knockout of Nur77 promoted the release of LDH after ox-LDL treatment (Figure 7(a)). Then, Western blotting analysis showed an obvious increase in the expression level of NLRP3 from Nur77−/− PMs compared with that in WT PMs after ox-LDL stimulation (Figures 7(b) and 7(f)). Simultaneously, we observed that the expression levels of the c-caspase-1 and c-IL-1*β* were further upregulated in Nur77−/− PMs after ox-LDL treatment compared with that in WT PMs (Figures 7(c), 7(d), 7(g), and 7(h)). These findings were in agreement with the results obtained in vivo. Besides, as shown in Figures 7(e) and 7(i), a significant upregulation in the protein expression of GSMD-N was also shown in Nur77−/− PMs after ox-LDL administration compared with that in WT PMs. These data suggest that Nur77 deletion deteriorates NLRP3 inflammasome-mediated inflammation and might also promote pyroptosis in PMs.

To identify the mechanism by which silence of Nur77 upregulated NLRP3 protein, we examined whether Nur77 bound directly to the NLRP3 gene promoter and suppressed its transcription. We cloned a luciferase-reporter driven by the promoter fragment of the mice NLRP3 gene and performed the promoter reporter assay in Raw264.7 cells. The luciferase assays showed that Nur77 significantly decreased NLRP3-luciferase activity, indicating that Nur77 transcriptionally inhibits the NLRP3 promoter (Figure 7(j)). Accordingly, the ChIP assay results revealed that Nur77 could bind to the promoter of NLRP3, and this binding might be enhanced following ox-LDL stimulation (Figure 7(k)). These results importantly indicate that Nur77 transcriptionally inhibits NLRP3 in macrophage through directly binding to the upstream promoter of NLRP3.

## 4. Discussion

Although previous studies have demonstrated that Nur77 is important in the progression of atherosclerosis, the role of Nur77 in atherogenesis remains vague. Here, we reported that Nur77 deficiency accelerated atherosclerotic plaque progression and destabilization in ApoE−/− mice. Mechanistically, Nur77 deficiency potently exacerbated macrophage NLRP3 inflammasome-mediated inflammation in vitro and in vivo.

To confirm the role of Nur77 in the atherogenesis, we first developed a mouse model of atherosclerosis through partially ligation of LCCA and LRA. Our results showed that larger and less stable atherosclerotic lesions characterized with reduced collagen content, macrophage infiltration, and lipid deposition 4 weeks after surgery in ApoE−/− Nur77−/− mice compared with ApoE−/− mice. Previous studies have reported that Nur77 was involved in the attenuation of the cardiac hypertrophy induced by *β*-adrenergic stimulation [21]. Nur77 also controlled vascular smooth muscle cell proliferation [22], migration [18], and reduced neointimal hyperplasia [23]. Recent study showed that Nur77 inhibited endothelial cell proliferation and angiogenesis and suppressed atherosclerotic lesion progression [24]. However, the functional role of Nur77 in macrophage seems to be paradoxical. Three laboratories previously have determined the effect of Nur77-deficient bone marrow on atherogenesis in LDLR−/− mice after bone marrow transplantation, and two of them revealed accelerated atherogenesis with higher macrophage content [14, 15], whereas one team observed no differences in plaque burden in comparison with WT bone marrow transplanted mice [25]. The discrepancy in the outcomes may be explained by the subtle differences in experimental setup, such as irradiation regime, recovery after transplantation, and duration of the high-fat diet [13]. Of note, our finding was in agreement with most published research results that deficiency of Nur77 amplified atherosclerosis development and destabilization. The limitation of our work is that the mice lack Nur77 throughout the body, and since Nur77 has been reported to also affect smooth muscle cells and endothelial cells, our model might be more complex.

Inflammation with macrophage infiltration is a key feature of atherosclerosis [5]. Targeting IL-1*β*-mediated inflammation using an IL-1*β*-neutralizing antibody canakinumab reduces the risk of cardiovascular events in patients [8]. The NLRP3 inflammasome contains the ASC that activates pro-caspase-1 into c-caspase-1, which catalyzes the cleavage of the precursor cytokine pro-IL-1*β* into mature, active proinflammatory cytokine c-IL-1*β* [6]. Duewell et al. first reported that bone marrow deficiency of NLRP3 and IL-1*β* significantly slowed the progression of atherosclerotic plaques compared with low-density lipoprotein receptor (LDLR)−/− mice transplanted with wild-type bone marrow [26]. Consistent with previous works [27], our result showed that high expressions of NLRP3 inflammasome components such as c-caspase-1 and c-IL-1*β* were observed in carotid atherosclerosis. In vitro, ox-LDL activated the NLRP3 inflammasome in PMs, probably by interfering with lysosomal function [26]. Moreover, it is recently discovered that c-caspase-1 not only processes the pro-IL-1*β* into their mature forms but also mediates proteolytic cleavage of GSMD that is responsible for pyroptosis [5, 28]. Our result revealed that the expression level of GSMD-N was obviously increased after high concentration of ox-LDL stimulation, suggesting pyroptosis might also be involved. Additional experiments are needed to verify these possibilities.

Another significant finding was that Nur77 deficiency significantly exacerbated macrophage NLRP3 inflammasome-mediated inflammation in atheroma. Consistent with the previous studies [29, 30], our results showed that Nur77 was highly expressed in macrophages in the context of atherosclerosis. As a transcription factor, Nur77 functions as regulating several cellular processes, including cell growth, activation, proliferation, and apoptosis [13]. Studies have also shown that in macrophage overexpression of Nur77 could reduce inflammatory factor expression of IL-1*β*, IL-6, and IL-8 [29, 31]. Recently, Hanna et al. and Hamers et al. demonstrated that the deficiency of Nur77 was associated with macrophage polarization towards the proinflammatory phenotype [14, 15]. However, the function role of Nur77 in NLRP3 inflammasome of atheroma has not been elucidated. In present study, we firstly demonstrated that Nur77 deficiency significantly promoted NLRP3-mediated inflammatory responses, thus contributing to the development of atherosclerosis. The activation of NLRP3 inflammasome usually requires two independent signals, that is, priming and activation, and the key priming event is the transcriptional upregulation of NLRP3 via NF-*κ*B signaling [5]. Here, we firstly reported that Nur77 negatively regulated NLRP3 inflammasome activity by inhibiting NLRP3 transcription. This findings established a new paradigm for the biological effects of Nur77 and might have major implications for treating atherosclerosis.

## 5. Conclusion

**Figure 8:**
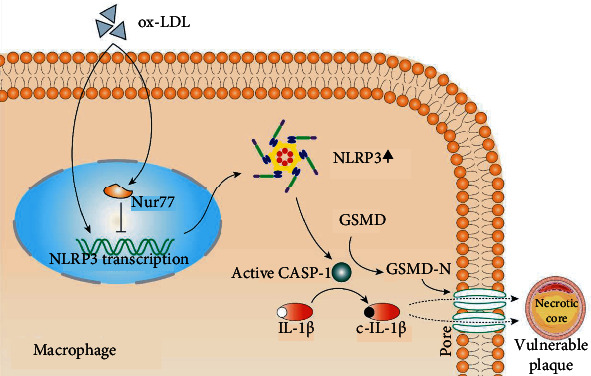
Graphic illustration of the role of Nur77 in NLRP3 inflammasome-mediated inflammation and atherogenesis. Nur77 directly transcriptionally inhibits NLRP3 expression and attenuates macrophage NLRP3 inflammasome-mediated inflammatory responses, thereby slowing atherosclerotic plaque progression.

In summary, we have demonstrated that the deficiency of Nur77 increased atherosclerotic plaque burden. This detrimental effect was accompanied by a deterioration of NLRP3-mediated inflammation in macrophages (Figure 8). Our findings provide direct evidence that Nur77 plays an important role in regulating NLRP3 inflammasome and thus represents a promising target for the treatment of atherosclerosis.

## Figures and Tables

**Table 1:** Lesion features in ApoE−/− mice and ApoE−/− Nur77−/− mice.

Mice	Vulnerable phenotype	Introplaque haemorrhage	Rupture with thrombus	Multilayer with discontinuity
ApoE-/- (*n* = 10)	40% (4)	30% (3)	20% (2)	30% (3)
ApoE-/- Nur77-/- (*n* = 10)	90% (9)^∗^	60% (6)	40% (4)	70% (7)^∗^

^∗^ *P* < 0.05, significantly different from ApoE−/− group.

## Data Availability

The data used to support the findings of this study are included within the article.

## Supplementary Materials

Supplementary Materials Supplemental Figure S1: the effect of Nur77 deficiency on serum lipid profiles and hepatorenal function. Triglyceride (A), total cholesterol (B), HDL (C), LDL (D), alanine aminotransferase (E), and creatinine (F) levels were detected in serum samples in mice from each group 4 weeks after operations (*n* = 11). TG = triglyceride; TC = total cholesterol; HDL = high-density lipoprotein cholesterol; LDL = low-density lipoprotein cholesterol; ALT = alanine aminotransferase; Cr = creatinine. Values are shown as the mean ± SEM. No significant differences were observed among the groups. ^∗^*P* < 0.05.
