# Supplementary material for: Nur77 Deficiency Exacerbates Macrophage NLRP3 Inflammasome-Mediated Inflammation and Accelerates Atherosclerosis
Source: Oxid Med Cell Longev. 2022 Apr 13;2022:2017815. doi: 10.1155/2022/2017815 (PMC9020982; doi:10.1155/2022/2017815)
Supplement: Supplementary Materials — Supplemental Figure S1: the effect of Nur77 deficiency on serum lipid profiles and hepatorenal function. Triglyceride (A), total cholesterol (B), HDL (C), LDL (D), alanine aminotransferase (E), and creatinine (F) levels were detected in serum samples in mice from each group 4 weeks after operations (n = 11). TG = triglyceride; TC = total cholesterol; HDL = high-density lipoprotein cholesterol; LDL = low-density lipoprotein cholesterol; ALT = alanine aminotransferase; Cr = creatinine. Values are shown as the mean ± SEM. No significant differences were observed among the groups. ∗P < 0.05. [file 2017815.f1.docx]

# Nur77 deficiency exacerbates macrophages NLRP3 inflammasome-mediatedinflammation and accelerates atherosclerosis

Supplemental Figures 1

#
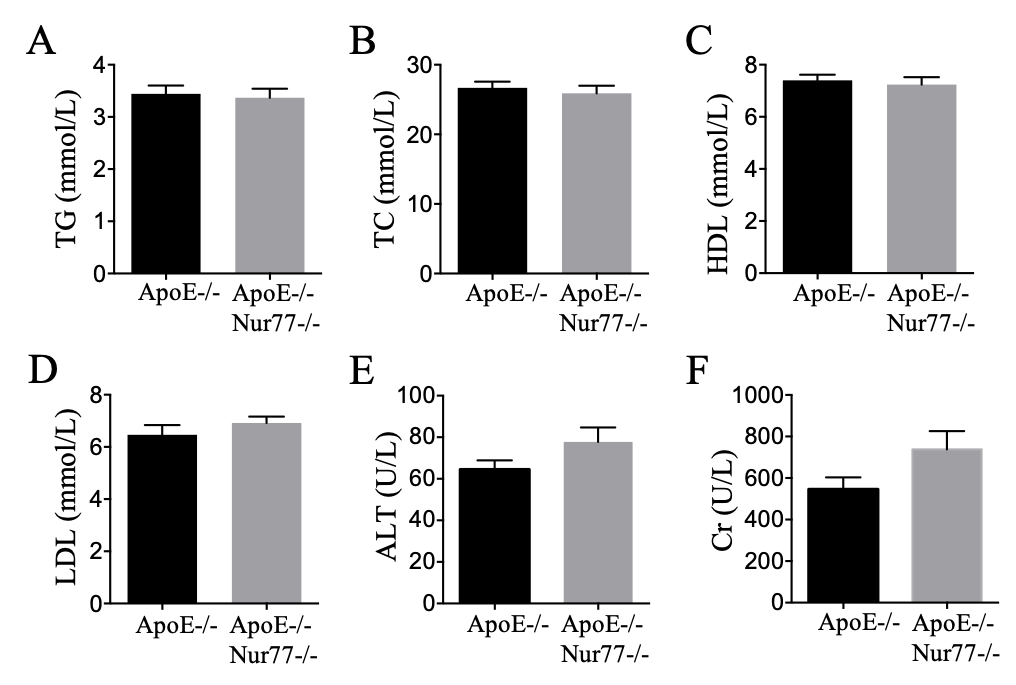
Supplemental figures

**Supplemental Figure S1:** Theeffectof Nur77 deficiencyon serum lipid profiles and hepatorenal function.Triglyceride (A), total cholesterol (B), HDL (C), LDL (D), Alanine aminotransferase (E), Creatinine (F)levels were detected in serum samples in mice from each group 4 weeks after operations (n = 11). TG = triglyceride, TC = total cholesterol, HDL = high density lipoprotein cholesterol, LDL = low density lipoprotein cholesterol, ALT = Alanine aminotransferase, Cr = creatinine. Values are shown as the mean ± SEM. No significant differences were observed among the groups.**P*< 0.05.
